# Discovering cooperative traits in crop plants

**DOI:** 10.1371/journal.pbio.3001892

**Published:** 2022-11-30

**Authors:** Susan A. Dudley

**Affiliations:** Department of Biology, McMaster University, Hamilton, Ontario, Canada

## Abstract

Plants should cooperate, but do they? What does plant cooperation look like? This Primer explores the implications of a study in PLOS Biology that demonstrates a practical and powerful methodology for exploring plant cooperation.

## Teaser

Plants should cooperate, but do they? What does plant cooperation look like? A study in *PLOS Biology* demonstrates a practical and powerful methodology for exploring plant cooperation.

Complex, dense, plant neighborhoods that may include plants of other species, unrelated plants of the same species, and relatives provide opportunities for competition and cooperation among plants. The problem of whether and how plants of the same species cooperate is one of interest to evolutionary biologists, ecologists, and crop breeders. In his formative papers, W. D. Hamilton [1] theorized that cooperative and even costly altruistic behaviors can evolve if these behaviors benefit relatives, which is now called kin selection theory. Hamilton recognized that plants have the opportunity for kin selection. The most used model of competition and cooperation in plants is the “Tragedy of the Commons,” where the most fit individuals within a stand “cheat” and compete strongly, but the best performing stands are populations of weak competitors [2]. In this model, competition is assumed the major interaction in plants growing together, and cooperation is increased when neighbors compete poorly. Selecting for weak competitors in crop breeding has increased yield [3], but identifying candidate traits for cooperation is difficult. A new study published in *PLOS Biology* by Wuest and colleagues [4] develops a powerful methodology for screening plants for cooperative loci and traits.

This methodology builds on an older approach of experimentally manipulating plant genotypes into siblings (hereafter, kin) or non-siblings of the same species (hereafter, strangers). Groups of kin or stranger plants in greenhouse pots provide microcosms with major evolutionary and ecological processes at play. Many kin versus stranger studies examined whether within-species biodiversity benefits plants [5]. Plants all need light, water, and mineral nutrients, so neighboring plants that compete for different pools of these resources should have higher performance. Kin are genetically and phenotypically more similar than strangers, so siblings should compete more strongly than strangers. If genetic diversity in competing plants reduces their impact on each other, then families should have higher average performance in diverse stranger groups than uniform kin groups.

Other studies explored kin selection in plants [5]. If plants can direct cooperation towards relatives, then plants growing with siblings should have higher fitness. Potentially, kin selection will favor constitutive altruism in populations in which all neighbors are highly related [1]. Such indiscriminate altruism should not increase the average fitness of kin relative to strangers as individuals would be equally cooperative or competitive with kin and strangers [1].

This simple approach of comparing average fitness of sibling and stranger groups has found that in some studies, plants had higher average fitness when growing with kin, in others plants had lower fitness when growing with kin, and for nearly half, it did not matter [5]. While these results are intriguing, suggesting both kin selection and niche partitioning can occur in plants, this approach of determining the average fitness consequences of growing with siblings and strangers does not indicate what alleles and traits allow kin selection and niche partitioning.

Though many of the early papers implicitly assumed that plants were oblivious of their surroundings, scientists now know that plants sense and respond to many aspects of the environment, including the presence of neighbors [6,7]. Using this insight, my lab and other have approached kin selection by asking how plants behave with kin and strangers. We measure the possible competitive traits of plants in sibling or stranger groups from the same set of families grown in common conditions [8,9]. These trait comparisons have shown that plants can sense and respond to the relatedness of neighbors, i.e., plants demonstrate kin recognition. They provide insight into potential cooperative and competitive traits. However, the test of cooperation is weak because it is based on one comparison between the average kin and the average stranger value. When the result does not match expectations, it could indicate that plants are not cooperating with kin, or just that plants are competing differently than predicted.

**Fig 1:**
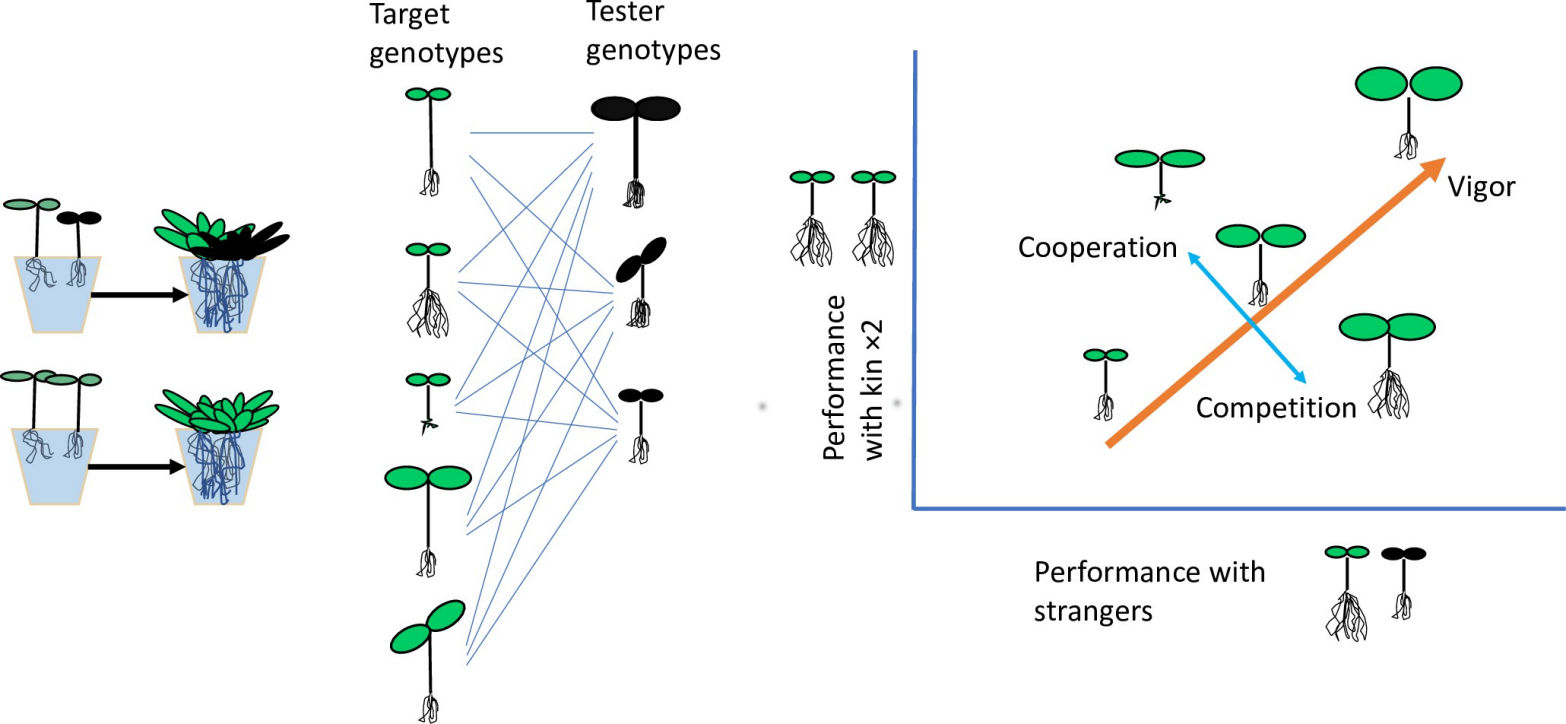
Screening for cooperative and competitive genotypes in Wuest and colleagues [4]. Seedlings vary in putative competitive traits: leaf size as a measure of plant size, stem elongation and leaf orientation as aboveground competitive traits, and root allocation as a belowground competitive trait. (A) The focal *Arabidopsis* genotypes (green) were paired with either kin, e.g., same genotype, or a stranger from the test genotypes (black), grown until mature, and the focal plant aboveground biomass harvested for performance. (B) Since all 98 focal genotypes were grown with each of 10 tester genotypes, the average genotype performance with a stranger also provides a relative measure of competitive ability [10]. (C) Each point is a focal genotype, with the average performance of 2 kin seedlings plotted against the average performance of a single seedling grown with tester genotypes. Genotypes with large seedling leaves grow into larger plants whether their competitor is kin or stranger, i.e., they vary in vigor (orange, thick line). Genotypes with similar vigor may perform with kin or with strangers, i.e., they vary in cooperation relative to competition (cyan, thin line). Here, the cooperation-competition associated variation, corresponding to the G-I axis in Fig 1 of Wuest and colleagues [4], is associated with root allocation. More cooperative genotypes have less root allocation, while genotypes with greater root allocation outcompete their neighbors.

The screening approach (Fig 1) in the study by Wuest and colleagues [4] incorporates the approach of growing plants with siblings and strangers and measuring performance (Fig 1A), but greatly increases its power. The crucial innovation the authors brought to this approach was to examine the patterns of variation among 98 genotypes of *Arabidopsis thaliana* in kin and stranger conditions, providing much more information than a simple comparison of mean performance or trait between kin and strangers. A more subtle innovation was made in creating the stranger conditions. By growing each focal genotype with the same 10 common tester genotypes (Fig 1B), they obtained a relative measure of competitive ability used in plant competitive ecology [10]. The third critical innovation was that their analysis of performance with kin and strangers provides 2 new traits (Fig 1C). The dominant but less interesting trait was vigor, as some genotypes just did well and others poorly whether they were grown with kin or strangers. But independently of vigor, some genotypes did better with kin, while others did better with strangers. This new trait measures cooperation versus competitiveness.

In contrast to previous kin selection–driven studies of how kin and strangers perform in competition, Wuest and colleagues focused on broadly screening for cooperation without preconceptions on where and why it would be found. Having found cooperative genotypes, they then could use the genetic tools developed in the *Arabidopsis* model system to identify a locus potentially associated with cooperation and further explore its phenotypic associations. An intriguing result is that root allocation was correlated with cooperation, in agreement with many studies finding reduced root allocation in kin compared to strangers [11]. The cooperative allele is also associated with disease resistance, indicating that the cooperation may be maintained as latent variation in populations with weak kin selection but strong selection for disease resistance.

Wuest and colleagues [4] have created a practical research program to screen for cooperative loci and traits that can be readily applied to any set of crop genotypes, following the example they demonstrated on *Arabidopsis*. Working in a model system with genetic tools will be particularly powerful, but in wild plants, researchers can explore correlations of cooperativeness with candidate cooperative and competitive traits. In crops, the approach could readily incorporate another competitive condition, that of crops with weeds, to screen for plants that are competitive with other species but cooperative with kin. This research approach is appropriate for any taxon, e.g., bacteria and fungi that can be easily manipulated into ecologically realistic, single genotype, and mixed genotype conditions. Kin selection has been most investigated in animals where our intuitions provide hypotheses about whether behaviors are cooperative. Here, we have a methodology to explore more hidden interactions.
